# Chondroitin/dermatan sulfate glycosyltransferase genes are essential for craniofacial development

**DOI:** 10.1371/journal.pgen.1010067

**Published:** 2022-02-22

**Authors:** Judith Habicher, Gaurav K. Varshney, Laura Waldmann, Daniel Snitting, Amin Allalou, Hanqing Zhang, Abdurrahman Ghanem, Caroline Öhman Mägi, Tabea Dierker, Lena Kjellén, Shawn M. Burgess, Johan Ledin

**Affiliations:** 1 Department of Organismal Biology, Uppsala University, Uppsala, Sweden; 2 Department of Cellular, Computational and Integrative Biology (CIBIO), University of Trento, Trento, Italy; 3 Genes & Human Disease Research Program, Oklahoma Medical Research Foundation, Oklahoma City, Oklahoma, United States of America; 4 Department of Information Technology, and SciLifeLab BioImage Informatics Facility, Uppsala University, Uppsala, Sweden; 5 Department of Immunology, Genetics and Pathology, Medical Genetics and Genomics, Uppsala University, Uppsala, Sweden; 6 Department for Medical Biochemistry and Microbiology, Uppsala University, Uppsala, Sweden; 7 Department for Engineering Sciences, Applied Materials Science, Uppsala University, Uppsala, Sweden; 8 Translational and Functional Genomics Branch, National Human Genome Research Institute, National Institutes of Health, Bethesda, Maryland, United States of America; University Hospital Ghent Center Medical Genetics: Universitair Ziekenhuis Gent Centrum Medische Genetica Gent, BELGIUM

## Abstract

Chondroitin/dermatan sulfate (CS/DS) proteoglycans are indispensable for animal development and homeostasis but the large number of enzymes involved in their biosynthesis have made CS/DS function a challenging problem to study genetically. In our study, we generated loss-of-function alleles in zebrafish genes encoding CS/DS biosynthetic enzymes and characterized the effect on development in single and double mutants. Homozygous mutants in *chsy1*, *csgalnact1a*, *csgalnat2*, *chpfa*, *ust* and *chst7*, respectively, develop to adults. However, *csgalnact1a*^*-/-*^ fish develop distinct craniofacial defects while the *chsy1*^*-/-*^ skeletal phenotype is milder and the remaining mutants display no gross morphological abnormalities. These results suggest a high redundancy for the CS/DS biosynthetic enzymes and to further reduce CS/DS biosynthesis we combined mutant alleles. The craniofacial phenotype is further enhanced in *csgalnact1a*^*-/-*^*;chsy1*^-/-^ adults and *csgalnact1a*^*-/-*^*;csgalnact2*^*-/-*^ larvae. While *csgalnact1a*^*-/-*^*;csgalnact2*^*-/-*^ was the most affected allele combination in our study, CS/DS is still not completely abolished. Transcriptome analysis of *chsy1*^*-/-*^, *csgalnact1a*^*-/-*^ *and csgalnact1a*^*-/-*^*;csgalnact2*^*-/-*^ larvae revealed that the expression had changed in a similar way in the three mutant lines but no differential expression was found in any of fifty GAG biosynthesis enzymes identified. Thus, zebrafish larvae do not increase transcription of GAG biosynthesis genes as a consequence of decreased CS/DS biosynthesis. The new zebrafish lines develop phenotypes similar to clinical characteristics of several human congenital disorders making the mutants potentially useful to study disease mechanisms and treatment.

## Author summary

The components of the extracellular matrix are crucial for interactions and communication between cells during animal development and disease progression. One major component of the extracellular matrix is chondroitin sulfate/dermatan sulfate (CS/DS) proteoglycans, which support and modify cell functions and tissue homeostasis. The biosynthesis of CS/DS is complex and no genetic models have been developed to specifically reduce CS/DS in the zebrafish model organism. We have used CRISPR/Cas9 technology to knock out key CS/DS biosynthesis genes. We find that knocking out single genes rarely causes major effects on zebrafish morphology and viability, but by combining several knockout alleles we could observe malformations in the zebrafish craniofacial skeleton. In addition, one combination of alleles was embryonic lethal. Our findings describe the role of CS/DS in the development of the head skeleton and give insights in the regulation of genes involved in CS/DS biosynthesis. The zebrafish mutants generated in this study can be used as tools to further study human diseases caused by mutations in CS/DS biosynthesis enzymes.

## Introduction

Heparan sulfate (HS) and chondroitin sulfate/dermatan sulfate (CS/DS) proteoglycans are heavily glycosylated proteins, crucial for animal development and homeostasis. They consist of long, unbranched, sulfated glycosaminoglycans (GAGs), covalently attached to core proteins via serine residues. GAGs are composed of repeating disaccharide units of an amino sugar and an hexuronic acid or galactose [1]. The GAG chains are synthesized in the Golgi apparatus, and the mature proteoglycans are secreted into the extracellular matrix, stored in secretory granules or are incorporated into the plasma membrane. In animals, GAGs are highly abundant and produced by most cells, where a single core protein may contain more than one type of GAG chain [1].

CS/DS proteogylcans are essential components of cartilage and bone tissues with important roles in development and function of the skeleton [2,3]. The pharyngeal cartilage is derived from migrating neural crest cells and produces an extracellular matrix rich in proteoglycans [4]. Most of these cartilage structures undergo endochondral ossification [5,6]. Mutations in the human genes encoding CS/DS biosynthesis enzymes cause a number of genetic disorders, often causing skeletal dysmorphism and growth retardation [7].

**Fig 1:**
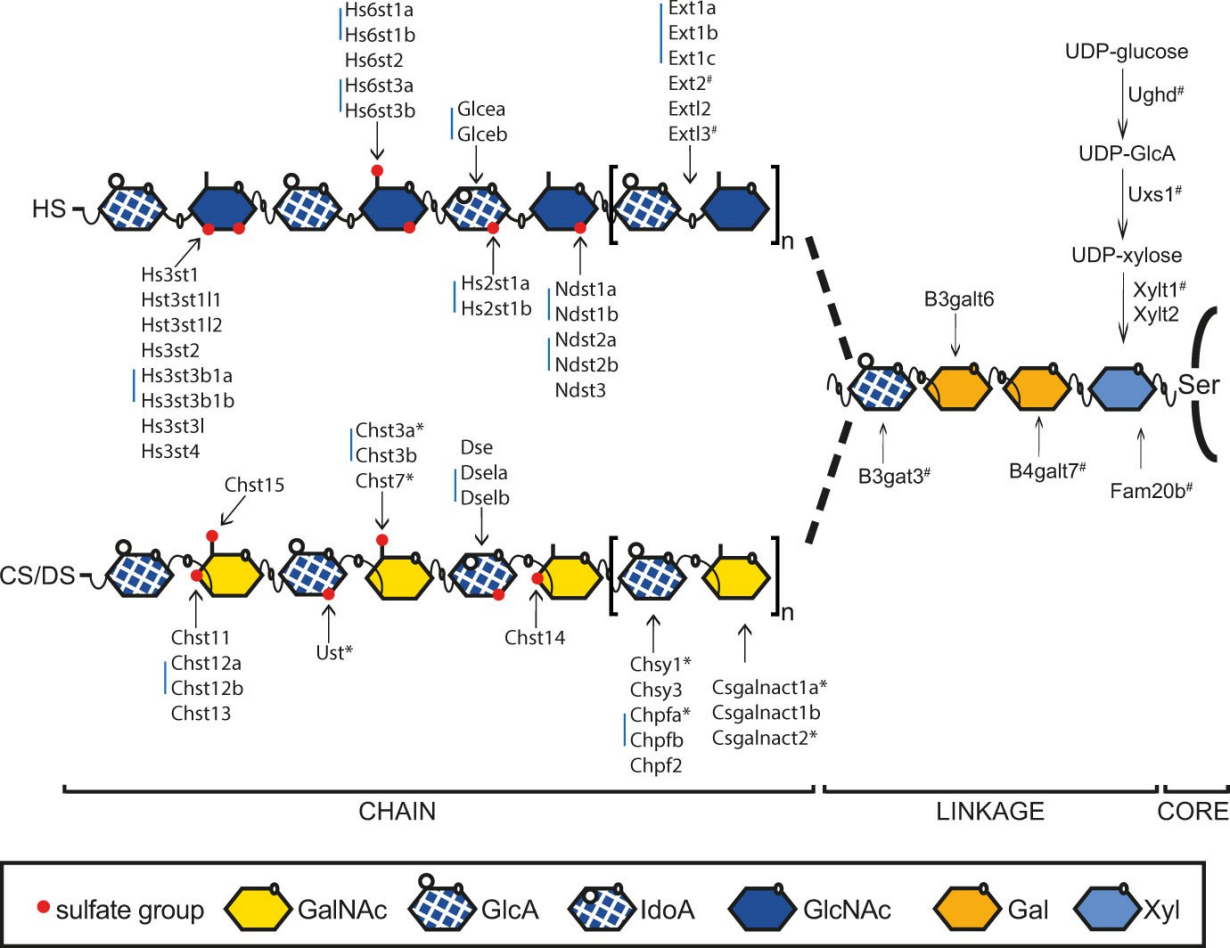
Heparan sulfate (HS) and chondroitin/dermatan sulfate (CS/DS) biosynthesis in zebrafish. HS and CS/DS chains are attached to serine (Ser) residues of the core protein. The first four monosaccharides form the linkage region of both HS and CS/DS. Extl3 initiates HS polymerization, while Csgalnact1a and Csgalnact2 perform this function in CS/DS polymerization. Elongation of HS is carried out by Ext enzymes, while Chsy and Chpf enzymes polymerize the CS/DS chain. The first modification of HS is carried out by Ndst enzymes, replacing an *N*-acetyl groups of GlcNAc residues with an *N*-sulfate group. Glcea and Glceb epimerize GlcA into IdoA in HS, while Dse, Dsela and Dselb are responsible for this modification in DS. Hs2st enzymes add a sulfate groups to the IdoA C-2 position of HS and Hs6st and Hs3st enzymes add sulfate groups to the GlcNAc or GlcNS residues. Ust adds a sulfate group to the hexuronic acid C-2 position of CS/DS while Chst11, Chst12a, Chst12b, Chst13 and Chst14 are GalNAc 4-O-sulfotransferases while Chst3a, Chst3b, Chst7 and Chst15 are GalNAc 6-O-sulfostransferases. Blue bars indicate two or three zebrafish genes orthologous to a single mammalian gene. Ndst3 in zebrafish is a single gene orthologous to two mammalian genes (NDST3 and NDST4). # Indicates previously published mutant zebrafish alleles while * indicates the targeted mutations reported in this study. Xyl: xylose, Gal: galactose, GlcNAc: *N*-acetylglucosamine, IdoA: iduronic acid, GlcA: glucuronic acid, GalNAc: *N*-acetylgalactosamine.

A complex biosynthetic machinery with a variety of enzymes is required to polymerize and modify CS/DS chains in vertebrates (Fig 1). Initiation of the CS/DS chain, on the tetrasaccharide linkage region which is shared between HS and CS/DS, is carried out by Csgalnact1 and Csgalncat2, while further polymerization is performed by Chsy and Chpf enzymes (Fig 1). Then the CS/DS chain is modified by epimerization and sulfation resulting in generation of binding sites for a vast number of proteins, including cytokines and chemokines, growth factors and morphogens, fibrous proteins like collagens, signaling receptors and cell adhesion proteins [8–10].

Zebrafish (*Danio rerio*) belonging to the teleost lineage, is a well-established animal model broadly used in biomedical research because of its genetic tractability. Teleosts underwent an additional round of whole genome duplication, compared to mammals and while most of the duplication was eventually lost through “rediplodization”, roughly 20% of zebrafish genes still have two copies in the genome. Zebrafish orthologues of mammalian HS and CS/DS biosynthetic enzymes have been previously identified (Fig 1) [11,12]. In some cases, two gene copies of GAG biosynthetic enzymes are retained (indicated by blue bars in Fig 1). In zebrafish the CS/DS biosynthetic enzymes are spatially and temporally regulated during embryonic development, indicating that tissue specific GAG structures exist [13–21].

The introduction of random mutations in the zebrafish genome has for a long time been used for forward genetic screens, where the mutagens can introduce loss-of-function mutations. A number of genetic mutants with defective GAG biosynthesis have been identified using this approach. Loss-of-function mutations in *ughd*, *uxs1*, *xylt1*, *fam20b*, *b4galt7*, *b3gat3*, genes for precursor transport and synthesis of the shared linkage structure of HS and CS/DS GAGs (Fig 1, indicated by #) typically develop abnormal jaw and pharyngeal cartilage structures [22,23], see also recent review [12]), highlighting the importance for these genes in skeletal development.

Zebrafish lines with mutations in HS glycosyltransferases, which reduce HS but not CS/DS accumulation, also affect craniofacial cartilage formation and in addition pectoral fin development and axon sorting [11]. No genetic knockout of CS/DS biosynthesis enzymes or core proteins has yet been reported in zebrafish. In mouse *Csgalnact1*^*-/-*^ and *Chsy*^*-/-*^ animals have been reported to show skeletal phenotypes [24,25]. Morpholino knockdown in zebrafish is a transient alternative to genetic knockouts, but the outcome can differ significantly [26], either because of morpholino off target effects [27] or due to genetic compensation [28]. This highlights the need of genetic mutants to complement knockdown experiments [29]. In this study we investigate the role of CS/DS in zebrafish development and the importance of individual biosynthesis enzymes. We hypothesized that knockout of one or several glycosyltransferase genes would decrease CD/DS production and affect zebrafish development, allowing us to study the specific role of CS/DS in zebrafish development. For this purpose, we have generated loss-of-function alleles in genes encoding for zebrafish CS/DS biosynthetic enzymes. The generated genetic mutants revealed a functional redundancy for enzymes in CS/DS biosynthesis and by combining different null-alleles, we were able to create a set of lines differing in CS/DS production and displaying varying phenotypes.

## Results and discussion

### Mutagenesis of key CS/DS biosynthetic enzymes

To reduce CS/DS biosynthesis in zebrafish we generated loss-of-function alleles in a number of genes encoding enzymes with key roles in CS/DS biosynthesis. We selected a subset of CS/DS modifying enzymes and glycosyltransferases for targeted mutation where we from previous studies expected a key function in CS/DS biosynthesis [12] and where suitable CRISPR targets could be found. We have previously developed protocols for high-throughput CRISPR/Cas9 modification of the zebrafish genome [30,31]. CRISPR targets were designed in the early part of the coding region of the CS/DS glycosyltransferases *csgalnact1a*, *csgalnact2*, *chsy1*, *chpfa* and the CS/DS sulfotransferases *ust*, *chst3a*, and *chst7*. Using this method, we isolated 20 zebrafish alleles with frame shifts in the coding sequence (Table 1) in seven different genes related to CS/DS biosynthesis (Table 1).

**Table 1:** Genomic and amino acid sequence for identified loss-of-function alleles. CRISPR target sequences are underlined and the PAM site is colored blue. Aberrant protein sequence is colored in red. Stop codon is indicated by a star (*). § (position of amino acid sequence interruption)/(total number of amino acids in protein).

Gene/Allele		Position of mutation^§^	Position of stop codon relative to conserved functional motif
*csgalnact1a*	ENSDART00000059322		
1_1; -1bp 1_2; -1bp 1_3; -5bp	ATGGGGCTGACTCGTCATCCCGAGGAGAAGCCGGTG M G L T R H P E E K P V ATGGGGCTGACTCGTCA-CCCGAGGAGAAGCCGGTGA M G L T R H P RRSR* ATGGGGCTGACTCGTCATCC-GAGGAGAAGCCGGTGA M G L T R H P RRSR* ATGGGGCTGACTCGTC-----GAGGAGAAGCCGGTGAG M G L T R RGEAGE	214/580 aa 214/580 aa 212/580 aa	upstream of the conserved B4GT domain including the conserved WGGED motif at 493–497 aa specific for the β4-glycosyltransferase family.[20,32]
2_2; -5bp	ACGAGCCACATGCCCATTAACATTGTGCTGCCGCTG T S H M P I N I V L P L ACGAGCCACAT----TTAACATTGTGCTGCCG T S H I*	310/580aa	
*csgalnact2*	ENSDART00000087533		
4_1; -2bp 4_2; -10bp	GTCACCCTCTTCCGGCCGTTCGGGCCCCTCATGAA V T L F R P F G P L M K GTCACCCTC——CCGGCCGTTCGGGCCCCTCATGAAAG V T L PAVRAPHES GTCACCCT------------TTCGGGCCCCTCATGAAAG V T L SGPS*	250/540aa 250/540aa	upstream of the conserved B4GT domain including the conserved W(G/V)GED motif at 453–457 aa specific for the β4-glycosyltransferase family.[20,32]
*chsy1*	ENSDART00000104536		
5_1; +1bp 5_2; -5bp	GTCATGACCGCGCAGAAGTACCTGAATAACCGCGCC V M T A Q K Y L N N R A GTCATGACCGCGCAAGAAGTACCTGAATAACCGCGC V M T A Q EVPE* GTCATGACCG-----AAGTACCTGAATAACCGCGC V M T EVPE*	95/801aa 93/801aa	upstream of the conserved B3GT domain including the FMRADD motif at 164–172 aa specific for the β3-glycosyltransferase family.[20,32] upstream of the conserved B4GT domain including the conserved W(G/V)GED motif at 493–499 aa specific for the β4-glycosyltransferase family.[20,32]
6_3; -4bp 6_5; -5bp	AGGACCTGGGCCAAGACCATCCCGGGCAAGGTGGAGT R T W A K T I P G K V E AGGACCTGGGCCAAGACCATCCC----AAGGTGGAGT R T W A K T I P RWS AGGACCTGGGCCAAGACCATCCCGGG-----TGGAGTT R T W A K T I P G GV	114/801aa 115/801aa	
*chpfa*	ENSDART00000113847		
61_1; -11bp 61_2; -1bp 61_3; -2bp 61_4; -4bp 61_5; -1bp	TTCCCGCCGAGAATAATCCCGTATAAACCAGTCAAC F P P R I I P Y K P V K TTCCCGCCGAGA-----------TAAACCAGTCAA F P P R * TTCCCGCCGAGAA-AATCCCGTATAAACCAGTCAA F P P R KSRINQS TTCCCGCCGAGAA--ATCCCGTATAAACCAGTCAA F P P R NPV* TTCCCGCCGAGA----TCCCGTATAAACCAGTCAA F P P R SRINQSN TTCCCGCCGAGA-TAATCCCGTATAAACCAGTCAA F P P R *	83/768aa 83/768aa 83/768aa 83/768aa 83/768aa	upstream of all conserved motifs in the CHPF family after the transmembrane region, very early in the protein. [20,32]
*ust*	ENSDART00000007735		
17_1; -5bp	CTGCTCTTCTGCCTCGGCTCGCTCTTTTACCAGCTGAAC L L F C L G S L F Y Q L N CTGCTCTTCTGCCTCG----lCTCTTTTACCAGCTGAALLFCLALLPAE	54/407aa	upstream of the 5’-phosphosulfate binding motif (5’PSB) aa 104–110) and the 3’-phosphate binding motif (3’PB) aa 177–192 for PAPS[21,33]
*chst3a*	ENSDART00000154120		
63_2; +2bp	AAGCTGACTCTCCGACGGACGCAGGAGCATCCAGTGCCC K L T L R R T Q E H P V P AAGCTGACTCTCCGACGGAGATGCAGGAGCATCCAGTGC K L T L R R RCRSIQC	37/416aa	upstream of the 5’-phosphosulfate binding motif (5’PSB) aa 139–148) and the 3’-phosphate binding motif (3’PB) aa 239–255 for PAPS [21,34]
*chst7*	ENSDART00000154363		
67_1; +5bp 67_2; -4bp 67_3; -2bp	TACCCGGGGGACGCGGGCAGTTTACAGGGAGCA Y P G D A G S L Q G A TACCCGGGTCAGGGACTGCGGGCAGTTTACAGGGAG Y P G QGLRAVYRE TACCCG----ACGCGGGCAGTTTACAGGGAG Y P TRAVYRE TACCCGGG--ACGCGGGCAGTTTACAGGGAGC Y P G RGQFGTS	121/416aa 120/416aa 121/416aa	downstream of the 5’-phosphosulfate binding motif (5’PSB) aa 109–118) and upstream of the 3’-phosphate binding motif (3’PB) aa 250–265 for PAPS [21,34]

### Some GAG biosynthetic enzymes are not critical for zebrafish development

To find genes with key roles in CS/DS biosynthesis we screened for morphological defects in homozygous mutants in larvae at 6 days post fertilization (dpf) and in adults (Table 2). With the exception of *csgalnact1a*^*-/-*^ (discussed below), they all displayed overall normal morphology (Table 2). From these results we conclude that defective gene function of single GAG biosynthetic enzymes typically allows for normal zebrafish development. Given the many enzymes that can both polymerize and modify the CS/DS molecule (Fig 1), this finding suggests widespread redundancy among CS/DS biosynthesis genes and might explain why no loss-of-function alleles in genes encoding for CS/DS biosynthesis enzymes have ever been identified in forward genetic screens, particularly since most screens focused on larval phenotypes.

**Table 2:** *Homozygous mutants survive into adulthood*. List of identified alleles and observed general morphology phenotypes at 6 dpf and in adults. (-) = not determined. *In this paper referred to as csgalnact1a^-/-^, csgalnact2^-/-^ and chsy1^-/-^, respectively.

Gene	Allele	Major morphological abnormalities	
		6 dpf	adult
*csgalnact1a*	*csgalnact1a*^*uu1_1*^ *	No	Yes
*csgalnact2*	*csgalnact2*^uu4_1^ *csgalnact2*^uu4_2^*	No No	No No
*chsy1*	*chsy1*^uu5_1^*	No	No
*chpfa*	*chpfa* ^uu61_1^ *chpfa* ^uu61_3^ *chpfa* ^uu61_4^ *chpfa* ^uu61_5^	No No No No	No - No -
*ust*	*ust* ^uu17_1^	No	No
*chst3a*	*chst3a* ^uu63_2^	No	-
*chst7*	*chst7* ^uu67_1^ *chst7* ^uu67_2^	No No	- No

### *csgalnact1a*^-/-^ adults develop craniofacial malformations

**Fig 2:**
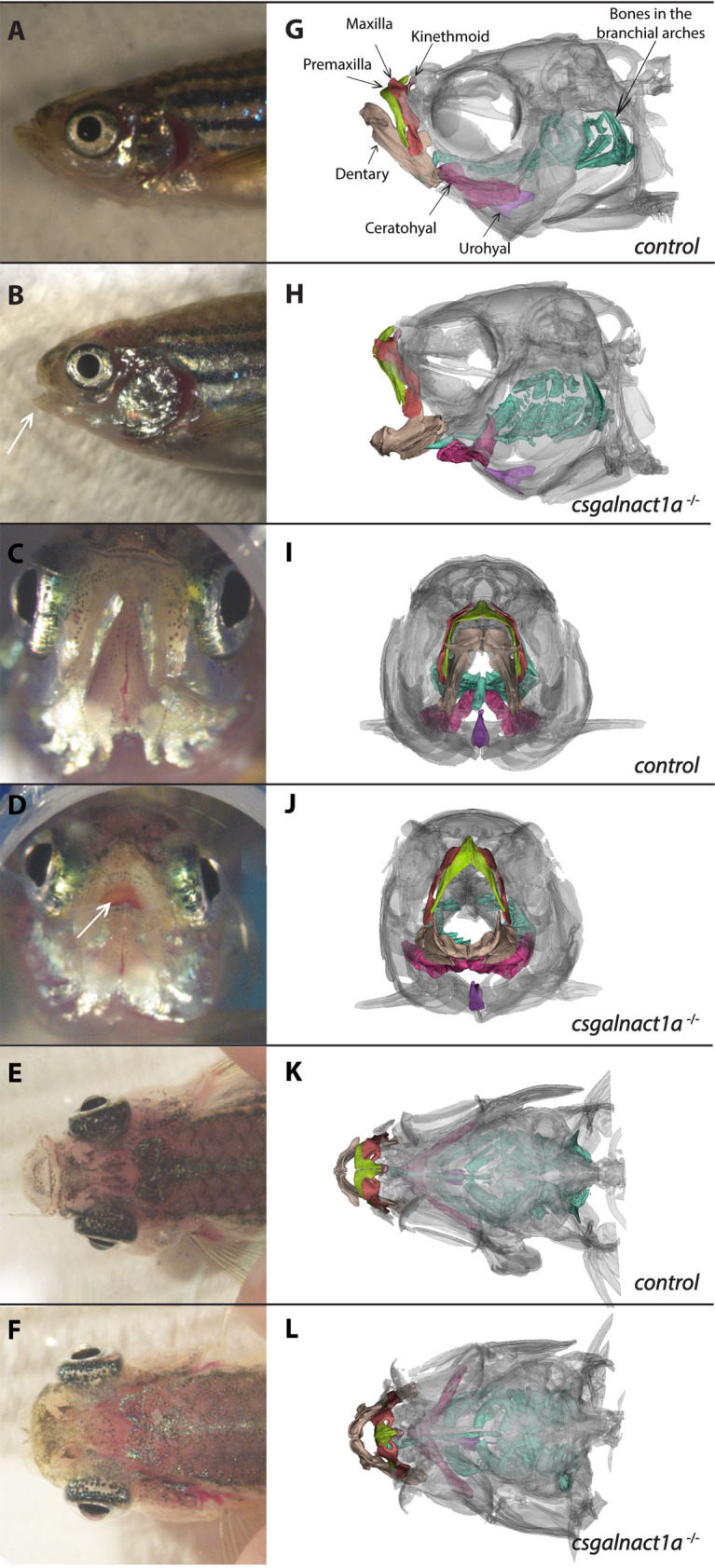
*Csgalnact1a*^-/-^ adults display obvious craniofacial malformations. Bright field images of zebrafish heads (A-F) and corresponding 3D reconstructions of microCT scans (G-H). Different skeletal elements are highlighted with colors. Compared to control adults (A,C,E,G,I,K), *csgalnact1a*^-/-^ adults (B,D,F,H,J,L) present a shortened jaw (arrow B) resulting in an open mouth phenotype (arrow D). In dorsal view the protruding eyes of *csgalnact1a*^-/-^ adults are apparent (F).

HS and CS/DS are synthesized on a common link structure and the committing step of CS/DS synthesis is the addition of a GalNAc residue by Csgalnact enzymes (Fig 1). Adult *csgalnact1a*^*-/-*^ zebrafish developed a fully penetrant head phenotype, compared to control animals, showing malformations of the head skeleton (Fig 2A–2F). To study head bone structures in detail we used micro-CT scanning to generate high resolution images of bone structures (Fig 2G–2L). Micro-CT scanning followed by 3D image reconstruction gave information on the relative position of adult cranial skeletal elements and made it possible to determine the size and shape of individual structures (S1 Movie and Fig 2G, 2I and 2K). The head of *csgalnact1a* mutants (Fig 2H, 2J and 2L) was wider and shorter than that of control adults, compressed along the anterior/posterior axis. The lower jaw, in particular the mandible element, was shortened and stouter in the adult *csgalnact1a* mutant (Fig 2H, arrow) compared to control adults (Fig 2G), resulting in a constantly open mouth (Fig 2J, arrow). Other morphological features of the *csgalnact1a* mutants were the protruding eyes and the widened angle of the ceratohyal element in mutants (Fig 2L). As a comparison, *Csgalnact1*^*-/-*^ mice showed impaired intramembranous ossification of the skull resulting in a shorter face, higher and broader calvaria, reminiscent of the zebrafish *csgalnact1a*^*-/-*^ phenotype [24]. In contrast to the zebrafish phenotype, the lower jaw in mice were largely unaffected. Human patients with mutations in *CSGALNACT1* have been described in several studies with mild skeletal dysplasia [35–37]. Our zebrafish *csgalnact1a*^*-/-*^ line may thus serve as a model to study this rare human syndrome (OMIM 618870).

### Combinatorial analysis of glycosyltransferase null-alleles result in a severe cranial skeletal phenotype

After addition of the first GalNAc residue to the linkage region by Csgalnact enzymes, the Chsy and Chpf enzymes polymerize CS/DS (Fig 1). Earlier published morpholino studies demonstrated a severe and embryonic lethal phenotype for *chsy1* morphants [38,39] suggesting that *chsy1* is critical for viability. In contrast, mouse *Chsy* mutants are slightly smaller than controls and viable albeit with joint patterning defects [25]. In several patients a mutation in *CHSY1* was found to be the cause for Temtamy Preaxial Brachydactyly syndrome (OMIM 605282) [39–42]. These patients show limb malformations, have a short stature and hearing loss.

**Fig 3:**
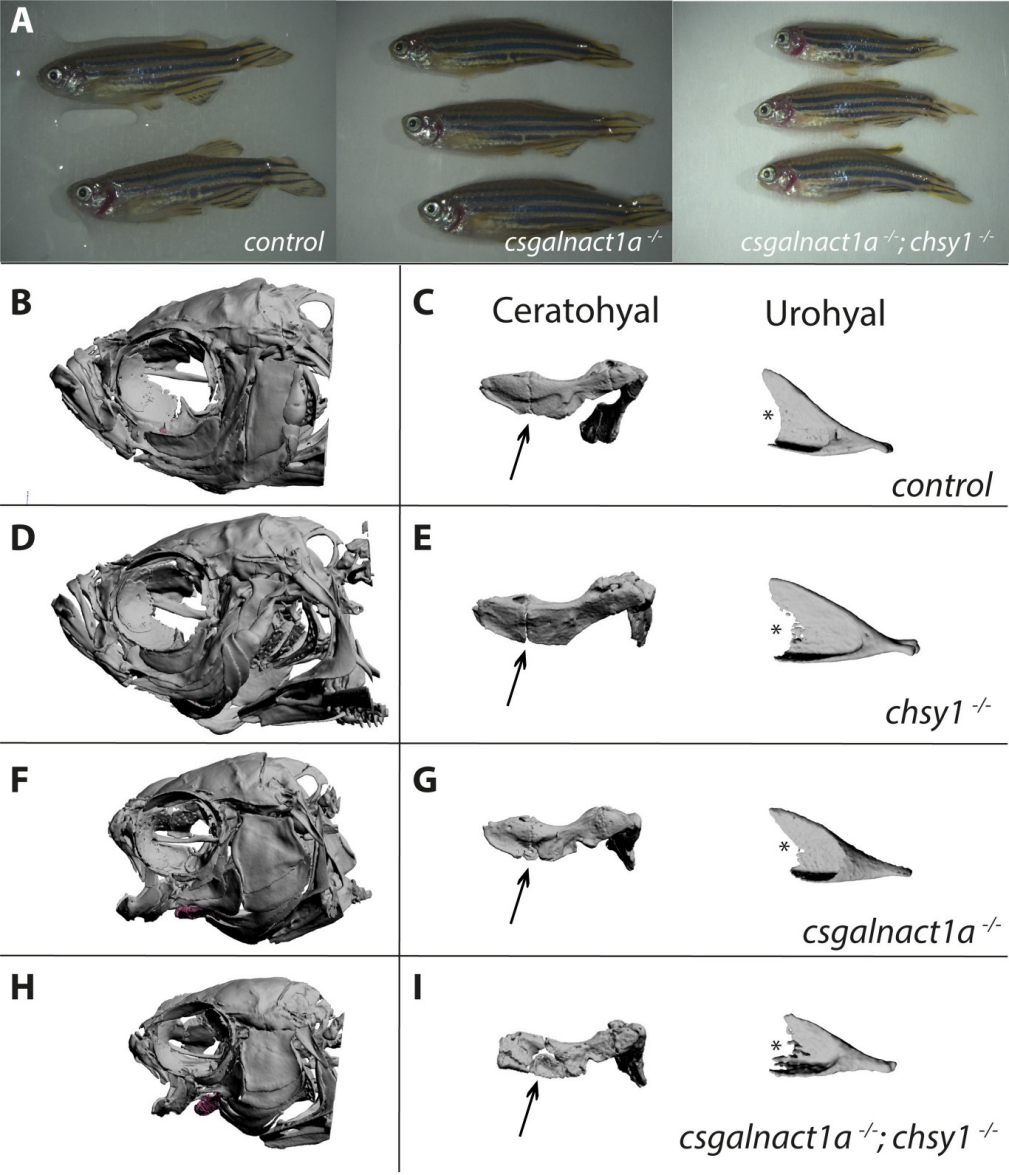
Severe malformation in the adult head skeleton. Alterations in the morphology of the ceratohyal and urohyal were magnified in all four genotypes (for spatial position of elements in the zebrafish head, see S1 movie). Adult *csgalnact1a*^*-/-*^ *; chsy1*^*-/-*^ zebrafish displayed gross malformations and reduced body size compared to control and *csgalnact1a*^-/-^ adults (A). 3D reconstructions of all genotypes showed that no bone structures were missing (B,D,F,H and S1–S4 Figs). However, shorter and misshaped elements were common in *csgalnact1a*^-/-^ (F) and *csgalnact1a*^*-/-*^ *; chsy1*^*-/-*^ fish (H), while the *chsy1*^*-/-*^ skeletal phenotype (D) was milder, compared to the control (B). The differences in morphological integrity of skeletal elements was evident from magnifications, for example the boundary between the anterior and the posterior part (arrow) of the ceratohyal and posterior edge (*) and of the urohyal (C,E,G,I and S5-12).

In contrast to the strong head phenotype in *csgalnact1a*^*-/-*^ adult zebrafish (Fig 3A, 3B and 3F), we found that *chsy1*^-/-^ zebrafish did not develop severe morphological defects (Fig 3D), more similar to the corresponding mouse knockouts than to the previously published morpholino studies [38,39]. To test if depletion of multiple CS/DS glycosyltransferases would enhance the phenotype compared to the single homozygous mutants, we then generated *csgalnact1a;chsy1* and *csgalnact1a;csgalnact2* double mutants. The *csgalnact1a;csgalnact2* double mutants were not adult viable, further discussed below, while *csgalnact1a*^*-/-*^*;chsy1*^*-/-*^ adults resulted in growth retardation and reduced body size compared to both control and *csgalnact1a*^*-/-*^ fish (Fig 3A and 3H). In addition we observed a disturbed swimming behavior and reduced buoyancy in *csgalnact1a;chsy1* double mutants. *csgalnact1a*^*-/-*^*;chsy1*^*-/-*^ adults developed a more severe skeletal phenotype compared to the *csgalnact1a*^*-/-*^ adults with a smaller head including the anterior parts of the skull (Fig 3H and 3F). Smooth, finished bone surface morphology in the ceratohyal as well as the urohyal and well-defined contours were characteristics of control adults (Fig 3C). In contrast, bone elements in mutant zebrafish were differently shaped and distorted and display irregular contours indicating a less dense bone volume (Fig 3E, 3G and 3I). The distinct boundary between the anterior and the posterior part of the ceratohyal (arrows) seen in controls (Fig 3C) was uneven in both *chsy1* (Fig 3E) and *csgalnact1a* (Fig 3G) mutants and almost absent in *csgalnact1a;chsy1* double mutants (Fig 3I). The midline segments, such as the urohyal, were compressed and showed an asymmetry in the midline. The posterior edge of the urohyal (*) was severely affected, which probably disturbs muscular attachment and therefore results in the constantly opened mouth. Notably, despite general gross malformations in the mutant lines, all cranial skeletal elements were present and morphologically distinct. This suggests that developmental patterning was generally intact but bone growth or formation was impacted. We conclude that the lack of both Csgalnact1a and Chsy1 has a more severe effect on adult mutant skeletal phenotypes than lack of either enzyme alone (Fig 3).

### *csgalnact1a*^*-/-*^ and *chsy1*^*-/-*^ develop malformations in the craniofacial cartilage elements already at larval stages

**Fig 4:**
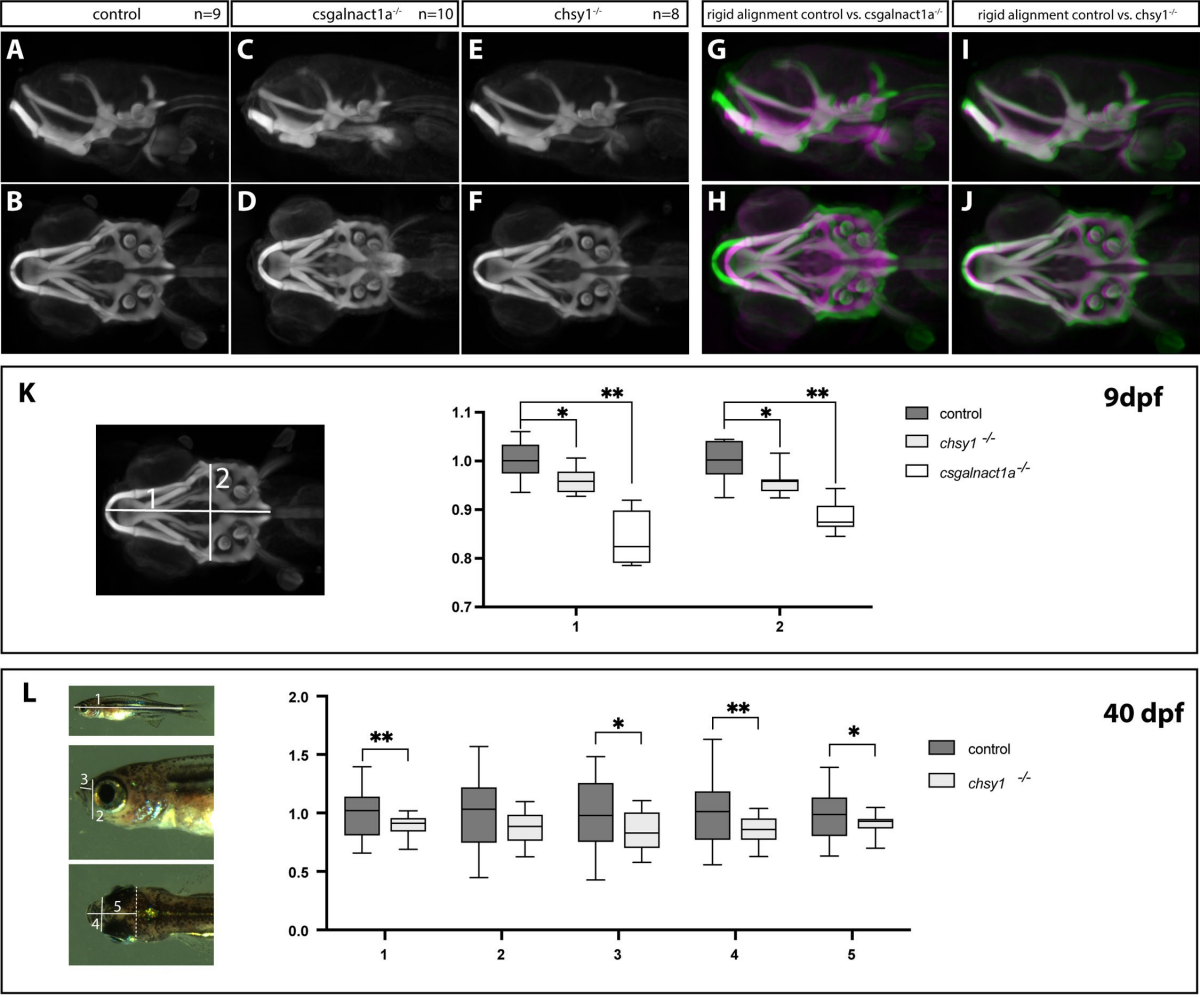
Malformations in the craniofacial cartilage structures. Maximum projections of average patterns generated from control, *csgalnact1a*^-/-^ and *chsy1*^-/-^ alcian blue stained larvae at 9 dpf show the pharyngeal cartilage structures in a ventral view (A-F). Maximum projections for each mutant (magenta) aligned to the control (green) are displayed using a rigid transformation (G-J). Measurements of the length and width of the head skeleton of 9 dpf larvae were performed on maximum projection images as shown in the image to the right (K) and plotted as a factor of control larvae (n = 8 for all genotype groups) (K). *csgalnact1a*^-/-^ and *chsy1*^-/-^ larval head skeleton is significantly smaller compared to control (K). Measurements of the standard body length (1) and different other measurements of the head (2–5) are indicated on images of 40 dpf old juvenile fish (L). *chsy1*^-/-^ juveniles are significantly shorter and have a smaller head compared to control larvae (chsy1^-/-^ n = 17, control n = 27) (L). Statistical significance is indicated by * for p-values <0.05 and ** for p-values <0.005.

The early skeletal structures in the zebrafish larvae are mainly cartilage. In order to visualize the skeleton already at developmental stages we performed alcian blue staining on csgalnact1a^-/-^, chsy1^-/-^ and their respective control larvae. Data from optical tomography where then used to generate maximum projections of average patterns resulting in an average 3D representation from 8–10 individuals of each genotype (Fig 4A–4F). These representations show that pharyngeal cartilage structures of mutant larvae at 9 dpf develop all major elements but that in particular csgalnact1a^-/-^ develop a malformed morphology (Fig 4C and 4D). By combining the 3D average structures of mutants with the controls, a rigid alignment showed the altered morphology more clearly as an overlay of mutant (magenta) and control (green) larvae was generated (Fig 4G–4J). The *chsy1*^*-/-*^ larvae developed a milder phenotype (Fig 4I–4J) compared to *csgalnact1a*^-/-^ larvae (Fig 4G–4H). The pharyngeal skeleton in *chsy1*^-/-^ larvae was smaller compared to control larvae and even further reduced in *csgalnact1a*^-/-^ larvae (Fig 4K). The mild skeletal phenotype of *chsy1*^-/-^ larvae was further analyzed in 40 dpf juveniles. The total body length and the head of *chsy1*^-/-^ juveniles were reduced compared to control fish, reminiscent of the facial dysmorphism and short stature in human patients [39–42].

### *csgalnact1a*^*-/-*^*;csgalnact2*^*-/-*^ develop malformations in the craniofacial cartilage elements at larval stages

**Fig 5:**
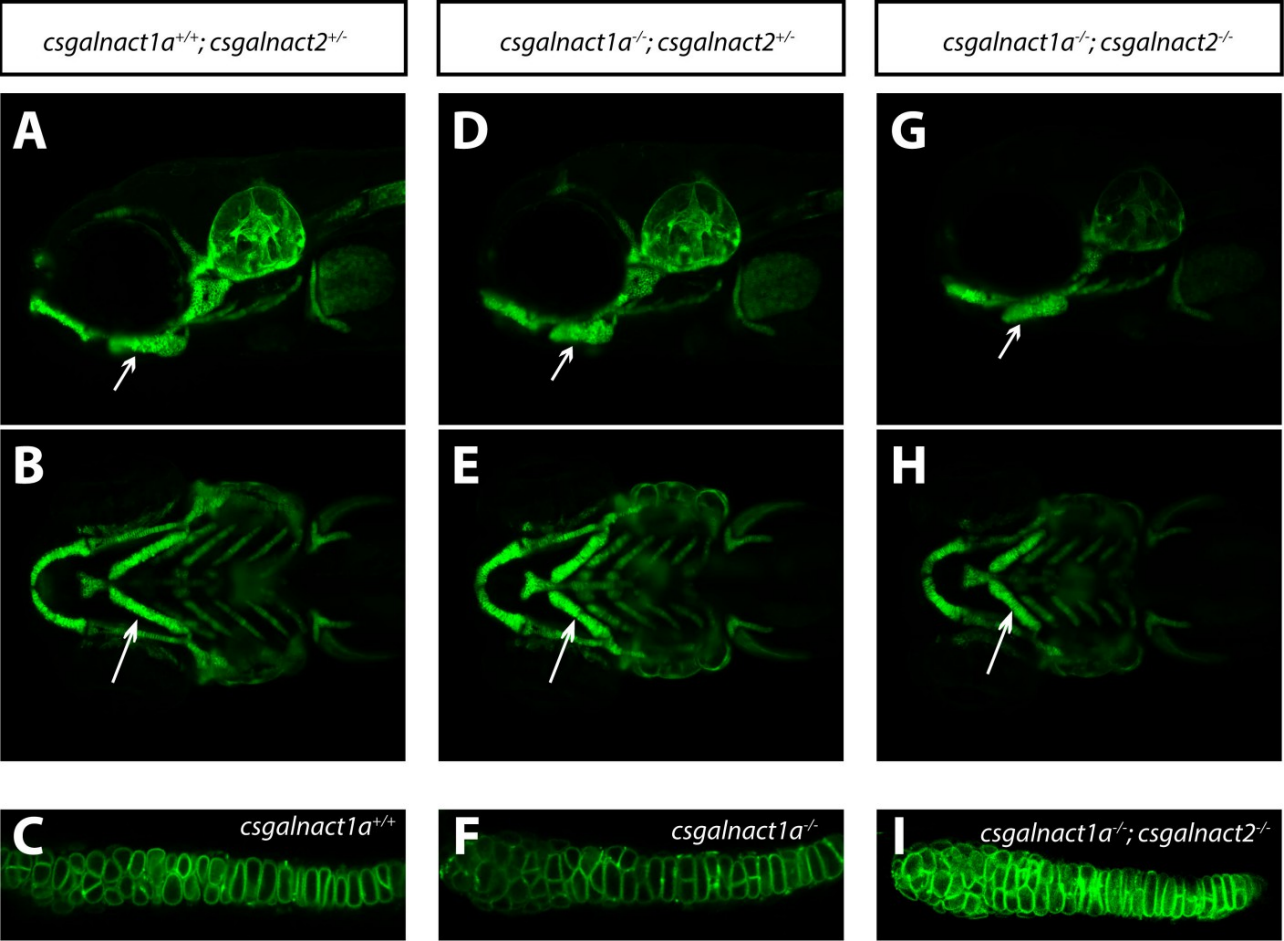
Craniofacial malformations in *csgalnact1a*^-/-^ and *csgalnact1a*^-/-^; *csgalnact2*^-/-^ larvae. LightSheet images of live *Tg(col2a1a*:*mEGFP)* larvae at 6 dpf show craniofacial cartilage elements formed in *csgalnact1a*^*+/+*^*;csgalnact2*^*+/-*^ (A,B,C), *csgalnact1a*^-/-^;*csgalnact2*^+/-^ (D,E,F) and csgalnact1a^-/-^;csgalnact2^-/-^ larvae (G,H,I). Lateral views (A,D,G) display a shorter ceratohyal, ventral views display a wider angle between the ceratohyal elements in *csgalnact1a*^*-/-*^*;csgalnact2*^*+/-*^ (E) and *csgalnact1a*^*-/-*^*;csgalnact2*^*-/-*^ (G) larvae (arrow) compared to controls (A,B). A detailed view on the chondorcytes within the ceratohyal (arrow) shows that intercalation occurs even in *csgalnact1a*^*-/-*^ (F) and *csgalnact1a*^*-/-*^*;csgalnact2*^*-/-*^ (I) larvae.

As mentioned above, removing *csgalnact1a* and *csgalnact2* together resulted in offspring not viable as adults, in contrast to *csgalnact2*^*-/-*^ fish that were phenotypically indistinguishable from controls (Table 2) and in contrast to *csgalnact1a*^*-/-*^*;chsy1*^*-/-*^ adults (Fig 3). At early larval stages *csgalnact1a* and *csgalnact2* showed strong expression in the craniofacial cartilage [20]. We investigated larvae in the transgenic *Tg(col2a1a*:mEGFP) background where the membranes of chondrocytes are labeled. At 6 dpf all craniofacial cartilage elements were formed in all larvae (Fig 5A–5H) and intercalation of chondrocytes occurred (Fig 5C, 5F and 5I). However, several elements were shorter and thicker in the *csgalnact1a*^*-/-*^ larvae (Fig 5D and 5E) compared to control larvae (Fig 5A and 5B). The ceratohyal was 15% shorter in *csgalnact1a*^*-/-*^ larvae compared to control larvae (p<0,005). The angle between the ceratohyal elements were widened in *csgalnact1a*^*-/-*^ larvae compared to controls. The craniofacial malformations were enhanced in *csgalnact1a*^*-/-*^*;csgalnact2*^*-/-*^ larvae (Fig 5G and 5H). *csgalnact1a*^*-/-*^*;csgalnact2*^*-/-*^ larvae also died before 10 dpf. Whether some CS/DS synthesis still occurred in these double mutants, and to what extent HS could be produced on core proteins normally modified by CS/DS, as suggested by previous experiments [15], remains to be investigated.

### Reduced CS/DS accumulation in glycosyltransferase mutants

**Fig 6:**
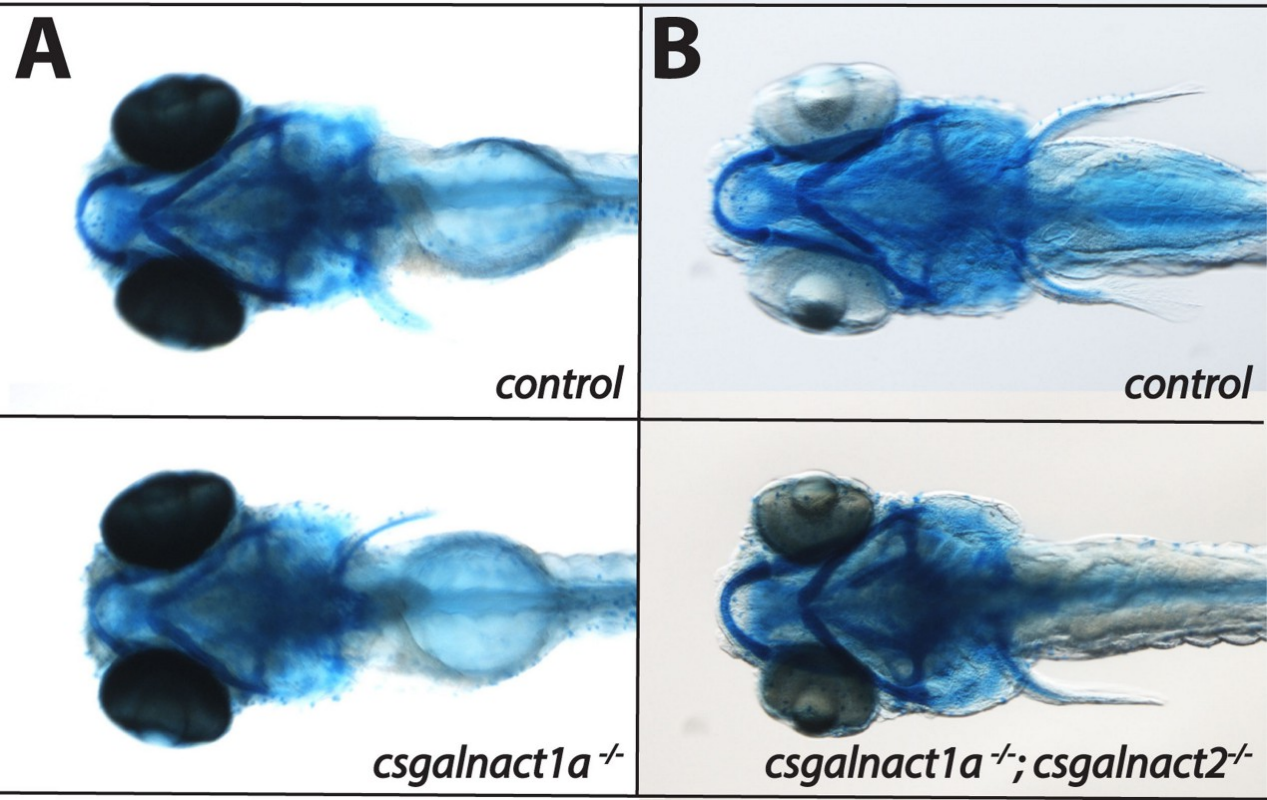
Larvae at 6 dpf were stained with alcian blue and are displayed in ventral views. No significant reduction of alcian blue staining was detected in either in the *csgalnact1a*^-/-^ (A) nor in the *csgalnact1a*^*-/-*^*;csgalnact2*^*-/-*^ (B) larvae.

**Fig 7:**
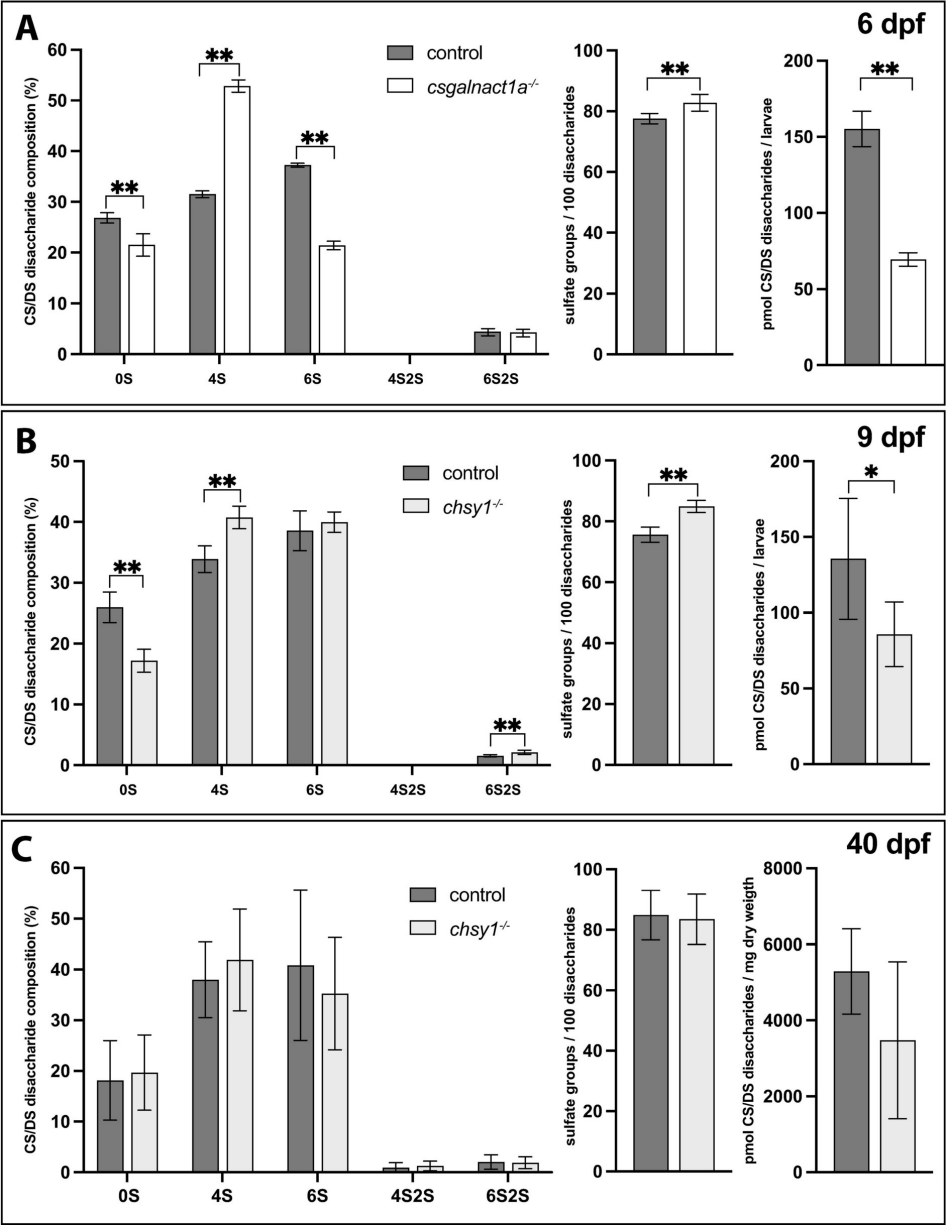
RPIP-HPLC analysis shows the CS/DS disaccharide composition (left charts), total sulfate group content/100 disaccharides (middle charts) and amount of CS/DS (right charts) for 6 dpf larvae (A), 9 dpf larvae (B) and 40 dpf juveniles (C). Statistical significances are indicated with * for p-values <0.05 and ** for p-values <0.005. Disaccharide species abbreviated as 0S (ΔHexA- GalNAc/ΔHexA-GlcNAc), 4S (ΔHexA-GalNAc4S), 6S (ΔHexA-GalNAc6S), 4S2S (ΔHexA2S-GalNAc4S), and 6S2S (ΔHexA2S-GalNAc6S).

We next investigated to what extent CS/DS glycosyltransefase mutants accumulate CS/DS. The Uxs1 (Fig 1) enzyme is necessary for GAG biosynthesis and 6 dpf *uxs1*^*-/-*^ zebrafish larvae have previously been shown to accumulate only 5% CS/DS compared to control larvae and do not stain with Alcian blue, which binds to the negatively charged sulfate groups of the carboxyl groups of hexuronic acids present in GAGs of the pharyngeal cartilage [15]. In contrast, the staining of *csgalnact1a*^-/-^ (Fig 6A) and *csgalnact1a*^*-/-*^*;csgalnact2*^*-/-*^ larvae (Fig 6B) is comparable to that of control larvae, indicating that sulfated GAGs are still accumulating in significant amounts, in particular in pharyngeal cartilage elements. We then analyzed the CS/DS disaccharide composition and amount in larvae with HPLC methodology [43]. In 6dpf *csgalnact1a*^-/-^ larvae the proportion of 4S disaccharides was distinctly increased at the expense of 6S disaccharides (Fig 7A). The content of sulfate groups per 100 disaccharides was slightly increased while the total CS/DS amount was decreased to less than half compared to control larvae (Fig 7A). A similar shift from 6S to 4S disaccharides was observed in a previous study in uxs1^-/-^ and b3gat3^-/-^ larvae [15] where defective linkage biosynthesis led to simultaneous HS and CS/DS reduction (Fig 1). This similarity may indicate a general property of CS/DS biosynthesis to shift from 6S to 4S when CS/DS polymerization is reduced. Alternatively, the reduction of CS/DS polymerization may be stronger in tissues which produce large amounts of CS/DS with higher 6S content–thereby making the overall CS/DS composition in mutant larvae resemble the 4S that dominated CS/DS of the earlier embryo [21]. The effect on CS/DS biosynthesis in 9 dpf *chsy1*^-/-^ was similar to the effect in *csgalnact1a*^-/-^ larvae (Fig 7B), with the notable exception that the proportion of 6S disaccharides in CS/DS was not changed compared to the control. The difference between effects in *chsy1*^-/-^ and *csgalnact1a*^-/-^ larvae may reflect different roles in the CS/DS biosynthesis or may be a consequence of differential expression of Chsy1 and Csgalnact1a during development [21]. An analysis of CS/DS in 40 dpf *chsy1*^-/-^ juveniles did not reveal statistically significant differences compared to control juveniles, suggesting that differences in CS/DS accumulation does not increase with age (Fig 7C). One possibility may be that older animals decrease CS/DS degradation in the extracellular matrix to compensate for reduced CS/DS biosynthesis. In addition, low amounts of non-sulfated, 4S, 6S and 4S2S disaccharides were detected in 6 dpf *csgalnact1a*^*-/-*^*;csgalnact2*^*-/-*^ and *csgalnact1a*^*-/-*^*;chsy1*^-/-^ larvae respectively. We conclude that none of the mutant lines presented in this study are completely devoid of CS/DS which emphasizes the redundant function of CS/DS biosynthesis enzymes. This confirms previous cell-based studies showing that while removal of one or two CS/DS glycosyltransferases reduce biosynthesis, the remaining enzymes can still to some extent polymerize CS/DS [44].

### Mutations in different CS/DS glycosyltransferases result in similar effects on transcriptome composition

We next investigated how a reduction in CS/DS biosynthesis affected the transcriptome in zebrafish larvae. We chose 6 dpf old larvae depleted of Chsy1, larvae depleted of Csgalnact1a and larvae depleted of Csgalnact1a and Csgalnact2 to include phenotypes ranging from mild to severe (Figs 4 and 5) for the transcriptome analysis.

**Fig 8:**
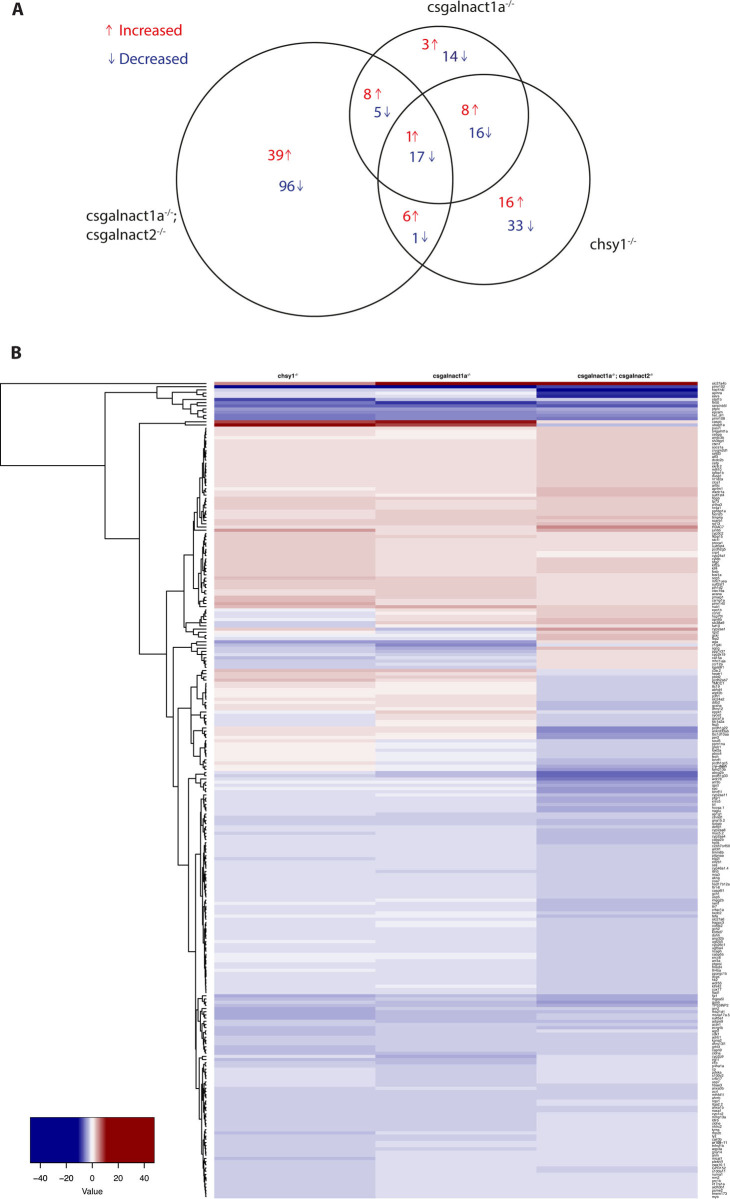
The area proportional venn diagram (A) shows differentially expressed genes in *csgalnact1a*^-/-^*, *csgalnact1a*^*-/-*^*;csgalnact2*^*-/-*^ and *chsy1*^*-/-*^ larvae compared to control larvae. The heat map (B) shows all genes with a two-fold increase (red) or decrease (blue) in the *csgalnact1a*^*-/-*^, *csgalnact1a*^*-/-*^*;csgalnact2*^*-/-*^ or *chsy1*^*-/-*^ larvae. * 50% of the *csgalnact1a*^*-/-*^ individuals lack one functional allele of chsy1.

In this study we defined differentially expression of a gene as a two-fold decrease or increase in gene expression (p<0.05). With this definition we detected 98 differentially expressed genes in larvae depleted of Chsy1 and 72 genes in Csgalnact1a depleted larvae. Approximately half of the genes were the same in the two groups (Fig 8A). Although both Chsy1 or Csgalnact1a are involved in CS/DS synthesis, we conclude that effects on the transcriptome of the larva in the respective mutants were not identical. The differences might be due to differential expression in the developing embryo since *csgalnact1a* expression is restricted mainly to developing cartilage structures while *chsy1* is more broadly expressed [38] and this difference is also manifested in the differences in skeletal phenotype (Figs 3 and 4). In larvae depleted of both Csgalnact1a and Csgalnact2, 173 genes were differently expressed compared to the control which was in line with the stronger and lethal phenotype (Fig 5). 18 genes were differently expressed in all three groups. In total 263 genes were differently expressed in at least one of the three mutant lines and the similarity is illustrated by heatmap analysis (Fig 8B). We grouped and colored these genes according to increase or decrease in transcription which revealed an overall striking similarity in the trend of change in expression between the mutant lines (i.e. if the expression of a gene is significantly changed in one mutant line, it is almost always changed in the same direction in the two other mutant lines) (Fig 8B). We conclude that the changes in transcriptome composition was similar but not identical in the three investigated cohorts of larvae.

### Mutations in *csgalnact1a*, *csgalnact2* or *chsy1* genes do not activate compensatory transcription of CS/DS glycosyltransferase genes

The fully viable and fertile adult *chsy1*^*-/-*^ phenotype was unexpected given the severe embryonic phenotype reported for *chsy1* morpholino knockdowns and distinct skeletal phenotype in patients with mutations the human ortholog CHSY1 [38,39]. The common observation of stronger morpholino knockdown phenotypes as compared to genetic knockout phenotypes has been discussed in recent years. One explanation may be that many reported morpholino phenotypes are at least in part a result of off-target effects [26]. Another possibility may be genetic compensation in mutants triggered by mutant mRNA degradation (i.e. nonsense-mediated decay) which induces compensatory transcription of genes with sequence similarities (i.e. paralogues) [28,45]. Candidates for genetic compensation in *chsy1*^*-/-*^ mutants would be other genes in the GAG biosynthesis machinery with high sequence similarity (Fig 1). However, all GAG glycosyltransferase genes identified on the microarray (*chsy1*, *chpf2*, *chpfa*, *chsy3*, *csgalnact1a* and *csgalnact2)* were expressed similarly in *chsy1*^*-/-*^ and control larvae, indicating that the induced frameshift mutations do not increase the degradation of glycosyltransferase mRNA (S2 Table). The same is true for *csgalnact1a*^*-/-*^ and *csgalnact1a*^-/-^*;csgalnact2*^*-/-*^ mutants (S2 Table). We further note that the reduced CS/DS biosynthesis in *chsy1*^*-/-*^, *csgalnact1a*^*-/-*^ and *csgalnact1a*^-/-^*;csgalnact2*^*-/-*^ larvae does not affect transcription of any GAG sulfotransferases, epimerases, sugar transporters and degrading enzymes. Thus, in the three mutant lines investigated, we found no evidence for genetic compensation due to mRNA decay. Our data further indicates that no mechanism exists to increase transcription of GAG biosynthesis genes as a consequence of missing CS/DS glycosyltransferases or decreased CS/DS accumulation.

### Aggrecan and Grhl3 are dysregulated in mutants

CS/DS proteoglycans are modified by CS/DS GAG chains which are critical for function. Aggrecan is a major structural component of the extracellular matrix and heavily modified by CS. Our transcriptome data from the *chsy1*^*-/-*^, *csgalnact1a*^*-/-*^ and *csgalnact1a*^-/-^*;csgalnact2*^*-/-*^ mutants revealed a small (≈2-fold) but significant upregulation of both *acana* and *acanb* (S2 Table), potentially representing a mechanism to compensate reduced CS/DS in the extracellular matrix. Our dataset revealed only small effects on the expression of 12 additional CS/DS proteoglycans, indicating that compensatory transcription of proteoglycan core proteins is not a general effect to meet reduced CS/DS polymerization. Another gene that stood out in our dataset was *grainyhead-like transcription factor 3* which was 2–3 fold downregulated in *chsy1*^*-/-*^, *csgalnact1a*^*-/-*^ and *csgalnact1a*^-/-^*;csgalnact2*^*-/-*^ larvae. This gene encodes a transcription factor which in humans has been linked to cleft lip and palate syndromes (OMIM 606713, [46]). In zebrafish, *grhl3* is expressed throughout the pharyngeal cartilage and morpholino knockdown of *grhl3* results in underdeveloped lower jaws [47]. Our results suggest that CS/DS is involved in activating *grhl3* expression but the mechanism remains to be investigated. One possibility is a reduced activity of upstream *grhl3* activators such as *irf6* and *p63*, which are slightly less expressed in CS/DS depleted larvae (S2 Table). Expression of *edn1*, *hand2* and *dlx3b*, genes in the downstream signaling pathway of *grhl3*, were not altered in the mutants analyzed (S2 Table).

## Conclusions

Despite having many important functions during animal development, mutants for CS/DS biosynthesis enzymes have never been isolated in forward genetic phenotypic screens in zebrafish. In this study, we have applied a CRISPR/Cas9-based reverse genetics approach to generate mutant alleles in a number of CS/DS biosynthesis genes. Our data show that elimination of single enzymes rarely causes major phenotypic changes, indicating a high degree of functional redundancy of CS/DS biosynthesis enzymes, which likely explains why mutant alleles in these genes have not been picked up in forward genetics phenotypic screens. To study the combinatorial effects of depleted CS/DS biosynthesis on zebrafish development we then combined multiple loss-of-function alleles of CS/DS enzymes which resulted in variable phenotypes ranging from subtle skeletal deformations in viable and fertile adults to severe morphological defects and lethality. Mutant fish that survived into adulthood predominantly developed malformations in the craniofacial skeleton. Transcriptome analysis of mutant zebrafish larvae with craniofacial phenotypes ranging from subtle to severe, reveal that expression of GAG biosynthesis genes is not affected which suggests that depleted CS/DS does not infer compensatory expression of other GAG biosynthesis genes. The analysis also showed that genes with an altered expression in one mutant typically were altered also in the other mutants, indicating that these genes are important for similar functions. We conclude that mutant alleles in CS/DS glycosyltransferase genes, alone and in combination, result in distinct effects on craniofacial skeleton and transcriptome composition and that the mutant lines will provide valuable tools for modeling diseases with a CS/DS component.

## Materials and methods

### Ethics statement

This study was approved by Animal Care and Use Committee of the NHGRI with the protocol number G-01-3 and by Uppsala Djurförsöksetiska nämnd, Uppsala, Sweden (Permit number C161/14 and 5.8.18-11830/2019).

### Target design

Using the CRISPR targets TrackHub in the USCS Genome Browser, two target sequences for each gene of interest, all 20 bp long, flanked by NGG, the protospacer adjacent motif (PAM), needed for target site recognition and cleavage by Cas9, were selected. Preferably, targets starting with GG, crucial for the T7 polymerase action, were preferred. In cases where suitable sgRNA recognition motifs (5’GG-N18-NGG3´) were not found, one or two Gs were incorporated at the beginning of the sgRNA, essentially as previously described [30]. We typically targeted sequences within the first or second transcribed exon and avoided target sites overlapping with regions of known DNA sequence variability [48]. Sequences of all targets are presented in Table 2.

### Preparation of sgRNAs and Cas9 RNA

Preparation of sgRNAs was carried out as previously described [49]. In short, annealing of a fragment containing the T7 promoter, the target specific sequence and a DNA stretch overlapping with the guide core sequence, with a second fragment containing the guide core sequence, was performed. The product was then used as a template for RNA *in vitro* transcription (HiScribe T7 High Yield RNA Synthesis Kit, NEB) and the generated RNA was purified prior to injection. To prepare Cas9 mRNA, pT3Ts-nCas9 plasmid (Addgene 46757, kindly provided by Dr. Wenbio Chen) was digested with Xba1 (NEB), purified and used for T3 driven *in vitro* transcription according to the manual provided by the manufacturer (mMESSAGE m MACHINE T3 Kit, Life Technologies). Integrity of the mRNA was confirmed on a denaturating gel and concentration was measured by Nanodrop.

### Animals

Animal husbandry procedures were performed according to the approved NHGRI animal protocol G-01-3. Animal experiments performed in Uppsala were approved by Uppsala Djurförsöksetiska nämnd, Uppsala, Sweden (Permit number C161/14). AB, TAB5 and Tg(*col2a1a*:mGFP)) [50] zebrafish were used.

### Injections

Fertilized zebrafish (TAB5) eggs were obtained in natural crosses, injected at the one-cell stage with 150ng/μl of Cas9 mRNA and 25ng/μl sgRNA per target in RNAse free H_2_O, as previously described [30].

### Genotyping by fluorescent PCR and sequencing

Injected founder fish were raised and outcrossed with wild-type zebrafish (TAB-5). Adult F1 fish were fin-clipped and PCR products were analyzed by fragment separation using capillary electrophoresis (or fragment length analysis), as previously described [51]. DNA extraction from either fin clip of adults or whole embryos was performed by dissolving tissue in 30μl 50mM NaOH for 20 min in 95°C, adding 60μl 50 mM Tris-HCl, and diluting 1:100. PCR (all primers listed in S1 Table) was performed, adding a third M13 forward primer, fluorescently labeled with 6-FAM. The length of the denaturated PCR products was measured on a Genetic Analyzer 3130xl using POP-7 polymer. The results were analyzed by Gene Mapper (Life Technologies) or Peak Scanner Software (Life Technologies), as previously described [31]. The length of the fragment/fragments was compared to the WT product length, predicted by in silico PCR using the USCS Genome browser. Heterozygous fish with two fragments, where deletions or insertions would predict a frame shift in the protein sequence (not a multiple of 3), were selected. These PCR products were cleaned up with ExoSAP-IT (Affymetrix), which degrades residual single-stranded primers and hydrolyzes remaining dNTPs. Due to addition of the M13 forward primer to the PCR reaction, M13 could now be used for sequencing all products. Sequences obtained for the heterozygous fish were aligned to the reference sequences with help of the web-based tool Poly Peak Parser [52]. Heterozygous carriers were incrossed after sequence confirmation. Their embryos were observed during early development, and raised.

### Imaging-lightsheet microscopy

Larvae were imaged by lightsheet microscopy in a Zeiss Z.1 microscope at 6 dpf. *Tg(col2a1a*:*mEGFP*) positive larvae were selected, anesthetized in 0,3% Tricaine solution and mounted in low melting agarose (1%) in FEB tubes. Z-stacks from different angles were taken and maximum intensity projections of lateral and ventral views were exported.

### Alcian blue

Larvae fixed in fresh 4% paraformaldehyde at 4°C overnight, were permeabilized using increasing concentrations of methanol and stored at -20°C. After washing (PBST), bleaching (30% hydrogen peroxide, 2 hours), and washing again (PBST) larvae were transferred into an Alcian blue solution (1% concentrated hydrochloric acid, 70% ethanol, 0.1% Alcian blue). Overnight incubation at room temperature was followed by rinsing with acidic ethanol (5% concentrated hydrochloric acid, 70% ethanol). Rehydration was performed in acidic ethanol of decreasing concentrations and finally samples were cleared in glycerol in PBST and imaged.

### GAG compositional analysis

CS/DS were isolated from zebrafish larvae and juveniles at 6 dpf (15 larvae/sample, 6 samples/genotype), 9 dpf (2 larva/sample, 6 samples/genotype) and 40 dpf (1 juvenile/sample, 5–6 samples/genotype). CS/DS was isolated from larvae and juveniles as described previously [53] and modified by [54]. CS/DS disaccharides generated after digestion with chondroitinase ABC and heparinase I, II, and III, respectively, were subjected to RPIP-HPLC analysis followed by post-column derivatization with cyanoacetamide and detection in a fluorescence detector [53].

### MicroCT

Eight adult zebrafish (2 controls, 3 *csgalnact1a*^-/-^; *chsy1*^+/+^ and 3 *csgalnact1a*^-/-^; *chsy1*^-/-^) were anesthetized with tricaine, fixed in fresh 4% paraformaldehyde, embedded in agarose and scanned using micro-computed tomography (μ-CT, Skyscan 1172, Bruker microCT, Kontich, Belgium). The scanner operated at a voltage of 80 kV and a current of 124 μm, with a 0.5 mm Al-filter. Images were acquired with an isotropic pixel size of 5.2 μm^2^. Reconstruction of cross-sections was done using software package NRecon (Bruker microCT, Kontich, Belgium). 3D reconstruction of the scan data was performed with Mimics Research 19.0 (Materialise software, Belgium) using a combination of manual and automatic threshold segmentation.

### Optical Projection Tomography (OPT)

For imaging 9 dpf fixed and alcian blue stained larvae were transferred in 99% Glycerol. OPT data was generated using the zOPT system described in [55] and generation of the average patterns are done using a similar method as described in [56]. Each group of 8–10 larvae was aligned to an average pattern using an Iterative Shape Averaging (ISA) algorithm [57]. All samples withing a group is first coarsely aligned to each other using a rigid alignment. From all the aligned images an average image is created. This average is then iteratively improved using an affine transformation and in the last iteration step a non-linear transformation. All average images are then positioned in the same view to make it easier to compare the group patterns.

### Gene expression analysis

Gene expression analysis was performed on samples of RNA isolated from 10–20 6 dpf larvae from clutches generated by separate crossings of adult zebrafish. Incross of *chsy1*^-/-^ produced chsy1 deficient offspring. Offspring from an incross of *csgalnact1a*^+/-^;*csgalnact2*^-/-^) produced offspring where the 25% of individuals deficient in both csgalnact1a and csgalnact2 developed a distinct phenotype (Fig 4E and 4F) and were isolated for analysis. Outcross of *csgalnact1a*^-/-^;*chsy1*^+/-^ with *csgalnact1a*^-/-^;*chsy1*^+/+^ produced offspring deficient in csgalnact1a (50% of offspring also lack one copy of chsy1). Three RNA samples of each group were isolated, in total 12 independent samples. 10–20 larvae were placed in 0,7 ml QIAzol (Qiagen), and total RNA was purified with the miRNeasy Mini kit (Qiagen) following the manufacturer’s instructions. RNA quality and quantity were ensured using Bioanalyzer (Agilent Technologies, Inc, Santa Clara, CA) and NanoDrop (Thermo Scientific, Wilmington, DE), respectively. Microarray analysis was performed at the NHGRI Microarray Core following the recommended Affymetrix protocol. Fragmented and labeled cDNA was hybridized onto GeneChip Human Gene 1.0 ST Arrays (Affymetrix, Santa Clara, CA). Staining of biotinylated cDNA and scanning of arrays were performed according to the manufacturer’s recommendations. The heatmap was created in R (version 3.5.1) using the heatmap.2 command (gplots package, version 3.0.1), with standard parameters. The dendrogram was computed using the complete linkage method to find similar clusters based on Euclidian distance.

## Supporting information

S1 Fig CT scanned and rendered skeletal structures in the wild-type control head skeleton. This interactive 3D PDF gives an overview as well as helps understanding the relation between the structures within the head. The ceratohyal is labeled and can be selected separately.
(PDF)

S2 Fig CT scanned and rendered skeletal structures in the *chsy*^-/-^ head skeleton. This interactive 3D PDF gives an overview as well as helps understanding the relation between the structures within the head. The ceratohyal is labeled and can be selected separately.
(PDF)

S3 Fig CT scanned and rendered skeletal structures in the *csgalnact1a*^-/-^ head skeleton. This interactive 3D PDF gives an overview as well as helps understanding the relation between the structures within the head. The ceratohyal is labeled and can be selected separately.
(PDF)

S4 Fig CT scanned and rendered skeletal structures in the *csgalnact1a*^-/-^; *chsy*^-/-^ head skeleton. This interactive 3D PDF gives an overview as well as helps understanding the relation between the structures within the head. The ceratohyal is labeled and can be selected separately.
(PDF)

S5 Fig This interactive 3D PDF shows the CT scanned and rendered ceratohyal of two wild-type control adults. (PDF)

S6 Fig This interactive 3D PDF shows the CT scanned and rendered ceratohyal of two *chsy*^-/-^ adults. (PDF)

S7 Fig This interactive 3D PDF shows the CT scanned and rendered ceratohyal of two *csgalnact1a*^-/-^ adults. (PDF)

S8 Fig This interactive 3D PDF shows the CT scanned and rendered ceratohyal of two *csgalnact1a*^-/-^; *chsy*^-/-^ adults. (PDF)

S9 Fig This interactive 3D PDF shows the CT scanned and rendered urohyal of two WT control adults. (PDF)

S10 Fig This interactive 3D PDF shows the CT scanned and rendered urohyal of two *chsy*^-/-^ adults. (PDF)

S11 Fig This interactive 3D PDF shows the CT scanned and rendered urohyal of two *csgalnact1a*^-/-^ adults. (PDF)

S12 Fig This interactive 3D PDF shows the CT scanned and rendered urohyal of two *csgalnact1a*^-/-^; *chsy*^-/-^ adults. (PDF)

S1 Table Forward and reverse primers for fluorescent fragment length analysis as well as sequencing. A M13 tag (TGTAAAACGACGGCCAGT) was added to the 5’ end of all forward primers and a PIG-tail tag (GTGTCTT) to the 5’ end of all reverse primers.
(DOCX)

S2 Table Microarray complete data set. The excel file contain three sheets: The sheet “Complete dataset” includes all gene expression data from the experiment. The sheet “GAG-biosynthesis enzymes” presents the gene expression of GAG glycosyl transferases and GAG-modifying enzymes identified in this experiment. The sheet “Proteoglycans” presents the gene expression of CS/DS proteoglycan core proteins identified in this experiment.
(XLSX)

S1 Movie CT scanned and rendered skeletal structures in the WT control head skeleton. This video gives an overview as well as helps understanding the relation between the ceratohyal (red) and the urohyal (blue) in relation tho the entire head skeleton.
(MOV)
