# Correction: Chondroitin/dermatan sulfate glycosyltransferase genes are essential for craniofacial development

**DOI:** 10.1371/journal.pgen.1010242

**Published:** 2022-05-24

**Authors:** The PLOS Genetics Staff

There are several errors in the formatting of Table 1. The publisher apologizes for the errors. The authors have provided a new version of Table 1 that preserves the original formatting as S1 File. Please view S1 File below.

## Supporting information

S1 File PDF copy of Table 1. This version of Table 1 preserves the original formatting.
(PDF)
