# Supplementary material for: Chondroitin/dermatan sulfate glycosyltransferase genes are essential for craniofacial development
Source: PLoS Genet. 2022 Feb 22;18(2):e1010067. doi: 10.1371/journal.pgen.1010067 (PMC8896900; doi:10.1371/journal.pgen.1010067)
Supplement: S1 Table — A M13 tag (TGTAAAACGACGGCCAGT) was added to the 5’ end of all forward primers and a PIG-tail tag (GTGTCTT) to the 5’ end of all reverse primers. (DOCX) [file pgen.1010067.s013.docx]

| **Gene** |  | **Forward Primer** | **Reverse Primer** |
| --- | --- | --- | --- |
| *csgalnact1* | Target 1 | TACCATGGTTTGGTGCATTG | ACCTGCAACAGTTTCTACGC |
|  | Target2 | CGGTTACCTGAAGTTGTGCAT | TGAGAAGGACAAGGGCTCTC |
| *csgalnact2* | Target 1 | GGCTTTCAGTGAAGGTCTGC | AGTGAATATGCGCTGGTTCC |
|  | Target2 | CACAAATGAAAGCCTCTCCA | CTACAGGACGGAGAGGGACA |
| *chsy1* | Target 1 | CGGAGGGATGATGATGATG | TTTGAGTGAACTGCCCCTTT |
|  | Target2 | TTGGACAGCGAGTTTCATCA | GACTTCTTCTGCGGAGGGTA |
| *chpfa* | Target 1 | CATCTTCGACCACTGCACGT | GAAAGGAGCCCGAAGACCAA |
|  | Target2 | CCATGGGGTATCTTGCGGTT | CGTCTTGAGTTGCGCAGTC |
| *chst3a* | Target 1 | TGGGTTCATCAGTTTAAGGGGT | TGTCCGTGTTGTGGCCATTA |
|  | Target2 | AGTGCCCTACAATGCTTCCA | CCTCTCCACATGCCAGAGTG |
| *chst7* | Target 1 | AATGAACGAGGTGCTCACGT | ACATATACCTCCACGCGACC |
|  | Target2 | GAACCGCCAAGTCCAAAACC | CCTGTTCAGGTGCGACTTCT |
| *ust* | Target 1 | GTGTCCGAACAGGAGAAAGC | GAGTCACGGCACAGATCGTA |
|  | Target2 | GTGTCCGAACAGGAGAAAGC | GTGTCCGAACAGGAGAAAGC |
| *M13-FAM* |  | TGTAAAACGACGGCCAGT |  |
