# Supplementary material for: Correction: Chondroitin/dermatan sulfate glycosyltransferase genes are essential for craniofacial development
Source: PLoS Genet. 2022 May 24;18(5):e1010242. doi: 10.1371/journal.pgen.1010242 (PMC9129007; doi:10.1371/journal.pgen.1010242)
Supplement: S1 File — This version of Table 1 preserves the original formatting. (PDF) [file pgen.1010242.s001.pdf]

Gene/Allele		Position of mutation §	Position of stop codon relative to conserved functional motif
csgalnact1a	ENSDART00000059322		
1_1; -1bp<br>1_2; -1bp<br>1_3; -5bp	ATGGGGCTGACTCGTCATCCCGAGGAGAAGCCGGTG<br>M G L T R H P E E K P V<br>ATGGGGCTGACTCGTCA-CCCGAGGAGAAGCCGGTGA<br>M G L T R H P R R S R *<br>ATGGGGCTGACTCGTCATCC-GAGGAGAAGCCGGTGA<br>M G L T R H P R R S R *<br>ATGGGGCTGACTCGTC-----GAGGAGAAGCCGGTGAG<br>M G L T R R G E A G E	214/580 aa<br>214/580 aa<br>212/580 aa	upstream of the conserved B4GT domain including the conserved WGGED motif at 493-497 aa specific for the β4-glycosyltransferase family.[20, 32]
2_2; -5bp	ACGAGCCACATGCCCATTAACATTGTGCTGCCGCTG<br>T S H M P I N I V L P L<br>ACGAGCCACAT-----TTAACATTGTGCTGCCG<br>T S H I *	310/580aa	
csgalnact2	ENSDART00000087533		
4_1; -2bp<br>4_2; -10bp	GTCACCCTCTTCCGGCCGTTCGGGCCCCTCATGAA<br>V T L F R P F G P L M K<br>GTCACCCTC--CCGGCCGTTCGGGCCCCTCATGAAAG<br>V T L P A V R A P H E S<br>GTCACCCT----------TTCGGGCCCCTCATGAAAG<br>V T L S G P S *	250/540aa<br>250/540aa	upstream of the conserved B4GT domain including the conserved W(G/V)GED motif at 453-457 aa specific for the β4-glycosyltransferase family.[20, 32]
chsy1	ENSDART00000104536		
5_1; +1bp<br>5_2; -5bp	GTCATGACCGCGCAGAAGTACCTGAATAACCGCGCC<br>V M T A Q K Y L N N R A<br>GTCATGACCGCGCAAGAAGTACCTGAATAACCGCGC<br>V M T A Q E V P E *<br>GTCATGACCG-----AAGTACCTGAATAACCGCGC<br>V M T E V P E *	95/801aa<br>93/801aa	upstream of the conserved B3GT domain including the FMRADD motif at 164-172 aa specific for the β3-glycosyltransferase family.[20, 32]
6_3; -4bp<br>6_5; -5bp	AGGACCTGGGCCAAGACCATCCCGGGCAAGGTGGAGT<br>R T W A K T I P G K V E<br>AGGACCTGGGCCAAGACCATCCC----AAGGTGGAGT<br>R T W A K T I P R W S<br>AGGACCTGGGCCAAGACCATCCCGGG-----TGGAGTT<br>R T W A K T I P G G V	114/801aa<br>115/801aa	upstream of the conserved B4GT domain including the conserved W(G/V)GED motif at 493-499 aa specific for the β4-glycosyltransferase family.[20, 32]
chpfa	ENSDART00000113847		
61_1; -11bp<br>61_2; -1bp<br>61_3; -2bp<br>61_4; -4bp<br>61_5; -1bp	TTCCCGCCGAGAATAATCCCGTATAAACCAGTCAAC<br>F P P R I I P Y K P V K<br>TTCCCGCCGAGA-----------TAAACCAGTCAA<br>F P P R *<br>TTCCCGCCGAGAA-AATCCCGTATAAACCAGTCAA<br>F P P R K S R I N Q S<br>TTCCCGCCGAGAA--ATCCCGTATAAACCAGTCAA<br>F P P R N P V *<br>TTCCCGCCGAGA----TCCCGTATAAACCAGTCAA<br>F P P R S R I N Q S N<br>TTCCCGCCGAGA-TAATCCCGTATAAACCAGTCAA<br>F P P R *	83/768aa<br>83/768aa<br>83/768aa<br>83/768aa<br>83/768aa	upstream of all conserved motifs in the CHPF family after the transmembrane region, very early in the protein. [20, 32]
ust	ENSDART00000007735		
17_1; -5bp	CTGCTCTTCTGCCTCGGCTCGCTCTTTTACCAGCTGAAC<br>L L F C L G S L F Y Q L N<br>CTGCTCTTCTGCCTCG-----CTCTTTTACCAGCTGAA<br>L L F C L A L L P A E	54/407aa	upstream of the 5'-phosphosulfate binding motif (5'PSB) aa 104-110) and the 3'-phosphate binding motif (3'PB) aa 177-192 for PAPS[21, 33]
chst3a	ENSDART00000154120		
63_2; +2bp	AAGCTGACTCTCCGACGGACGCAGGAGCATCCAGTGCCC<br>K L T L R R T Q E H P V P<br>AAGCTGACTCTCCGACGGAGATGCAGGAGCATCCAGTGC<br>K L T L R R R C R S I Q C	37/416aa	upstream of the 5'-phosphosulfate binding motif (5'PSB) aa 139-148) and the 3'-phosphate binding motif (3'PB) aa 239-255 for PAPS [21, 34]
chst7	ENSDART00000154363		
67_1; +5bp<br>67_2; -4bp<br>67_3; -2bp	TACCCGGGGGACGCGGGCAGTTTACAGGGAGCA<br>Y P G D A G S L Q G A<br>TACCCGGGTCAGGGACTGCGGGCAGTTTACAGGGAG<br>Y P G Q G L R A V Y R E<br>TACCCG----ACGCGGGCAGTTTACAGGGAG<br>Y P T R A V Y R E<br>TACCCGGG--ACGCGGGCAGTTTACAGGGAGC<br>Y P G R G Q F G T S	121/416aa<br>120/416aa<br>121/416aa	downstream of the 5'-phosphosulfate binding motif (5'PSB) aa 109-118) and upstream of the 3'-phosphate binding motif (3'PB) aa 250-265 for PAPS [21, 34]

Table 1. Genomic and amino acid sequence for identified loss-of-function alleles. CRISPR target sequences are underlined and the PAM site is colored blue. Aberrant protein sequence is colored in red. Stop codon is indicated by a star (*). § (position of amino acid sequence interruption)/(total number of amino acids in protein).
